# Microsatellite variation revealed panmictic pattern for *Triatoma brasiliensis* (Triatominae: Reduviidae) in rural northeastern Brazil: the control measures implications

**DOI:** 10.1186/s12863-020-00903-w

**Published:** 2020-08-27

**Authors:** Claudia Mendonça Bezerra, Carlota Josefovicz Belisário, Grasielle Caldas D’Ávilla Pessoa, Aline Cristine Luiz Rosa, Carla Patrícia Barezani, Flávio Campos Ferreira, Alberto Novaes Ramos, Ricardo Esteban Gürtler, Liléia Diotaiuti

**Affiliations:** 1 Departamento de Saúde Comunitária. Rua Professor Costa Mendes 1608 - Bloco Didático 5° andar - Rodolfo Teófilo, Universidade Federal do Ceará, Faculdade de Medicina, Fortaleza, Ceará CEP: 60430-140 Brazil; 2 Secretaria da Saúde do Estado do Ceará, Fortaleza, CE Brazil; 3 Grupo de Pesquisa em Triatomíneos e Epidemiologia da Doença de Chagas, Instituto René Rachou / FIOCRUZ – MG, Belo Horizonte, MG Brazil; 4 Universidade Federal de Minas Gerais, Belo Horizonte, MG Brazil; 5 Laboratory of Eco-Epidemiology, Universidad de Buenos Aires. Facultad de Ciencias Exactas y Naturales, Ciudad Universitaria, C1428EHA Buenos Aires, Argentina; 6 Instituto de Ecología, Genética y Evolución de Buenos Aires (IEGEBA). Consejo Nacional de Investigaciones Científicas y Técnicas-Universidad de Buenos Aires, Ciudad Universitaria, C1428EHA Buenos Aires, Argentina

**Keywords:** Chagas disease, *Triatoma brasiliensis*, Microsatellites, Genetic variability, Ceará state

## Abstract

**Background:**

*Triatoma brasiliensis* Neiva, 1911 is the main vector of *Trypanosoma cruzi* in the caatinga of Northeastern Brazil. Despite of its epidemiological relevance, there are few studies on its genetic variability. Using microsatellite markers, we characterized the variability and dynamics of infestation and reinfestation of *T. brasiliensis* after residual insecticide spraying in five surveys conducted in a well-defined rural area located in the municipality of Tauá, Ceará, between 2009 and 2015. We evaluated: (1) general variability among local of captures; (2) variability along the time analysis (2009, 2010 and 2015); (3) and reinfestation process.

**Results:**

On the analysis (1) global and pairwise *F*_ST_ values suggested absence of clusters among the area. AMOVA indicated that total variation is mainly represented by individual differences. Absence of clustering indicates a panmitic unit, with free gene flow. For (2), Pairwise *F*_ST_ indicated alterations in the genetic profile of the triatomines along the time. (3) Analysis of the reinfestation process showed that the domiciliary units investigated had different sources of infestation despite of its proximity.

**Conclusions:**

Observed homogeneity can be explained by the great dispersal capacity of *T. brasiliensis*, overlapping the different environments. Persistent house infestation in Tauá may be attributed to the occurrence of postspraying residual foci and the invasion of triatomines from their natural habitats.

## Background

Chagas disease is a chronic infection whose etiological agent is the protozoan *Trypanosoma cruzi* [1] (Protozoa, Sarcomastigophora, Kinetoplastida, Trypanosomatidae). It is considered a neglected disease by the World Health Organization and its transmission is closely associated with poverty [2, 3]. Currently, it is estimated that 5.7 million people are infected worldwide and other 70 million are at risk of acquiring the infection [2]. In Brazil, it is estimated that in 2015 there was between 1,4–3,4 million cases of *T. cruzi* infection, predominantly chronic cases with a high morbidity/mortality burden [4, 5].

Of the 65 species of triatomines described in Brazil, 28 species (43%) are found in the Northeastern region, and 20 of them (71%) are captured in human sleeping quarters (intradomiciles). The latter indicates these vectors’ capacity of frequent domiciliation [6–8]. Currently the *Triatoma brasiliensis* complex includes two subspecies (*Triatoma brasiliensis brasiliensis* and *Triatoma brasiliensis macromelasoma* [9] and six species (*Triatoma lenti*, *Triatoma juazeirensis*, *Triatoma melanica*, *Triatoma bahiensis* [10], *Triatoma sherlocki* [11] and *Triatoma petrocchiae* [8, 12]. *T. b. brasiliensis* Neiva, 1911, hereby referred as *T. brasiliensis,* is the main vector of *T. cruzi* in the *caatinga* region of Northeastern Brazil. It has a wide geographic distribution, high percentages of natural infection, great invasive potential and a prominent role in the domiciliary, peridomiciliary and sylvatic transmission cycles of *T. cruzi* [13–17]. In sylvatic environments, *T. brasiliensis* is usually found in crystalline rock outcrops, associated especially with rodents, marsupials and bats [18–20]. It is an aggressive and opportunistic species, with very eclectic host-feeding behavior; hence it can colonize several ecotopes in widely diverse environments [18, 21, 22].

Although *T. brasiliensis* is recognized as an epidemiologically important species, there are few studies on its genetic variability [23–27]. Among the available tools, microsatellite markers are highly useful because of their high genetic polymorphism [28]. Microsatellites or Short Tandem Repeats (STR) are small tandemly repeated DNA sequences (2–6 base pairs) that are widely found in coding and non-coding regions of the genome [29]. STR markers have a codominant inheritance pattern; it is a neutral marker, highly polymorphic and easily PCR-amplifiable. They are used to resolve issues of population structure, genetic diversity and conservation in animal populations [30, 31]. For triatomines, microsatellites have provided further insights into the sources of infestation and reinfestation of domiciliary habitats before and after application of residual insecticides [32–36]. In the northern Argentina, this tooll could determine putative sources of reinfestation of the *T. infestans* and showed that this dispersion is active and female biased.

Here, we characterize the distribution of genetic diversity of *T. brasiliensis* populations using microsatellite markers to: (a) determine the general variability among the samples obtained in 2009 and 2010 by local of capture; (b) observe the temporal variability in insects collected in 2009, 2010 and 2015; (c) investigate the reinfestation process in two domiciliary unities comparing the infestation in 2010 and 2015.

## Results

Six of the nine pairs of tested primers amplified microsatellite *loci*, but one of them (*locus* Tb8112) was monomorphic and hence was excluded from the analysis Primers described by Almeida et al. [23] could not amplify them.

### General variability

Differentiation between the 12 sites sampled in 2009 and 2010 accounted for only 0.76% of the total variability estimated by AMOVA. Among specimens at the same site the proportion was 19.80 and 79.43% among all sampled specimens. The *FST* value was 0.01 (*p* = 0.98), indicating gene flow between *T. brasiliensis* samples in the study area (Table 1). The pairwise *F*_ST_ values confirm the absence of clusters (Table 2), as found in the analysis of cluster using the software Structure. Mantel test revealed no association between genetic variability of samples and geographical distance. The genotyping result was detailed in Additional file 1.

**Table 1:** Analysis of molecular variance (AMOVA) for *Triatoma brasiliensis* using microsatellite of the: (I) samples obtained in 2009 and 2010 according local of capture; (II) insects collected in 2009, 2010 and 2015; (III) reinfestation process in Cachoeira do Júlio locality

	Source of variation	Variance components	Percentage of variation	Fixation Indices	P
**I**	**Among populations**	0.01 (Va)	0.76	0.01 (*F*_ST_)	0.98
	**Among individuals within populations**	0.36 (Vb)	19.80	0.20 (*F*_SC_)	0.00
	**Within individuals**	144.06 (Vc)	79.43	0.21 (*F*_CT_)	0.00
**II**	**Among populations**	0.00 (Va)	0.23	0.00 (*F*_ST_)	0.64
	**Among individuals within populations**	0.34 (Vb)	18.51	0.19 (*F*_SC_)	0.00
	**Within individuals**	149.23 (Vc)	81.26	0.19(*F*_CT_)	0.00
**III**	**Among populations**	0.05 (Va)	2.41	0.02 (*F*_ST_)	0.00
	**Among individuals within populations**	0.27 (Vb)	14.18	0.15 (*F*_SC_)	0.00
	**Within individuals**	159.09 (Vc)	83.41	0.17(*F*_CT_)	0.00

**Table 2:**
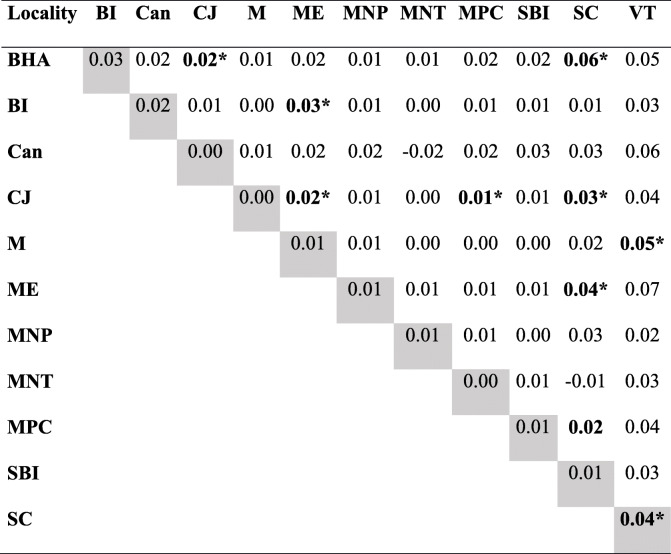
Pairwise *F*_ST_ values between the sites with *Triatoma brasiliensis* captures in Tauá, 2009 and 2010. Diagonally are the *F*_IS_ values

*BHA* Belo Horizonte do Alfredo, *BI* Benfinca do Incra, *Can* Canadá, *CJ* Cachoeira do Júlio, *M* Mutuca, *ME* Mutuca Evangelista, *MNP* Morada Nova do Pedro, *MNT* Morada Nova do Thomaz, *MPC* Mutuca Pedra da Cruz, *SBI* São Bento do Incra, *SC* São Cristóvão, *VT* Várzea do Touro.*significant values (*p* < 0.05)

Based on these results, the sample was treated as a single population and the vector survey periods were taken into consideration to assess infestation dynamics in Tauá.

### Variability along the time

The number of alleles per *locus* ranged from seven (Tb860) to 25 (Tb124), and the mean number per sample ranged from 10.60 (April 2010) to 13.40 (August 2015) (Table 3; Additional file 2). Observed and expected heterozygosity values are shown in Table 4. All samples showed Hardy-Weinberg disequilibrium as a result of heterozygosity deficit. The same result was found when the *loci* were evaluated, except for Tb860. The probability of occurrence of null alleles was low in all *loci*. The lowest frequencies were found in Tb860 and the higher in Tb728.

**Table 3:** Number of alleles per *locus* in *Triatoma brasiliensis* captured in Tauá

	Samples								
***Locus***	Feb/2009	Aug/2009	Apr/2010	Oct/2010	Aug/2015	Av	sd	N	bp
**Tb728**	8	9	9	9	10	9.00	0.71	10	300–318
**Tb830**	12	11	12	10	12	11.40	0.90	14	274–388
**Tb860**	6	6	6	5	6	5.80	0.45	7	390–400
**Tb8124**	21	16	22	14	20	18.60	3.44	25	214–268
**Tb7180**	15	15	18	15	19	16.40	1.95	21	218–260
**Mean**	12.40	11.40	13.40	10.60	13.40	12.24	1.24	15.40	
**s.d.**	5.94	4.16	6.54	4.04	5.98	5.33	1.15	7.50	

*Av* average among *loci*; *sd* standard desviation; *N* number total of alleles per locus; *bp* base pair size of fragment

Feb/2009: February/2009; Aug/2009: August/2009; Apr/2010: April/2010; Oct/2010: October/2010 and Aug/2015: August/2015

**Table 4:** Observed (Ho) and expected (He) heterozygosity, and null allele frequencies estimated per *locus* (NA) in *Triatoma brasiliensis* captured in Tauá

	Tb728			Tb830			Tb860			Tb8124			Tb7180		
	Ho	He	NA	Ho	He	NA	Ho	He	NA	Ho	He	NA	Ho	He	NA
**February/2009**	0.52*	**0.75**	0.16	0.59*	**0.80**	0.11	0.67	0.69	0.06	0.59*	**0.85**	0.14	0.68*	**0.85**	0.09
**August/2009**	0.55*	**0.80**	0.16	0.73	0.79	0.02	0.61	0.71	0.09	0.61*	**0.83**	0.11	0.72	0.87	0.08
**April/2010**	0.50*	**0.76**	0.17	0.50*	**0.74**	0.13	0.63	0.68	0.06	0.53*	**0.84**	0.16	0.76*	**0.86**	0.06
**October/2010**	0.58*	**0.83**	0.18	0.66*	**0.78**	0.06	0.65	0.66	0.01	0.51*	**0.86**	0.18	0.68*	**0.85**	0.09
**August/2015**	0.56*	**0.76**	0.14	0.70*	**0.78**	0.03	0.62	0.63	0.01	0.57*	**0.85**	0.15	0.67*	**0.85**	0.10

*significant value (*p* < 0.05)

Most of the variance determined by AMOVA was found between specimens (81.26%), followed by within-group (18.51%) and between-group variation, in which variability was only 0.23%. The fixation indices were: *F*_ST_ = 0.00 (*p* = 0.64) *F*_SC_ = 0.19 (p = 0.00); and *F*_CT_ = 0.19 (*p* = 0.00) (Table 1). The pairwise *F*_ST_ ranged from zero to 0.01. The group of the fourth survey (October 2010) was different from the first one. The 2015 sample was significantly different from that collected in February 2009 and from the two samples of 2010 (*p* < 0.05) (Table 4). The largest within-group variability (*F*is) was found in 2015 (0.82), and the lowest in October 2010 (0.43). The groups of the first, fourth and fifth surveys had *F*is with *p* < 0.05, indicating a population structure and deviation from Hardy-Weinberg equilibrium (Table 5).

**Table 5:**
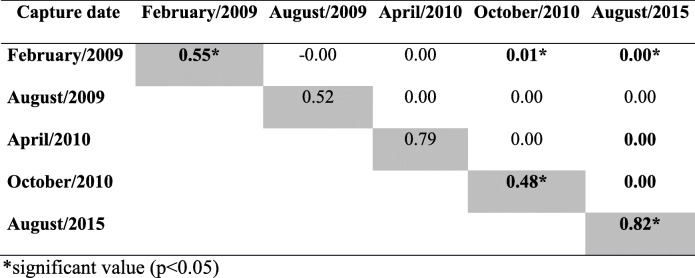
Pairwise *F*_ST_ (above diagonal) and *F*_IS_ (in diagonal) values genotypic of the *Triatoma brasiliensis* captured in Tauá from february 2009 to august 2015

*significant value (*p* < 0.05)

### Variability in Reinfestation in Cachoeira do Júlio

The number of alleles per *locus* ranged from five (Tb860) to 13 (Tb7180). The group that presented a greater mean number of alleles per *locus* was the one from the sylvatic environment (6.40) The lowest mean was from domiciliar unit (DU) 13 in 2015 (4.20) (Table 6; Additional file 3).

**Table 6:** Number of alleles per *locus* of *Triatoma brasiliensis* captured at a domiciliary unit in Cachoeira do Júlio locality

***Locus***	2010b_DU13	2010b_DU14	2015_DU13	2015_DU14	2015_W	Mean	s.d.	AN
**Tb728**	5	5	4	3	5	4.40	0.89	7
**Tb830**	6	6	4	6	7	5.80	1.10	10
**Tb860**	4	4	4	4	3	3.80	0.45	5
**Tb8124**	8	8	4	7	7	6.80	1.64	10
**Tb7180**	4	6	5	8	10	6.60	2.41	13
**Mean**	5.40	5.80	4.20	5.60	6.40	5.48	0.81	9.00
**s.d.**	1.67	1.48	0.45	2.07	2.61	1.66	0.80	3.08

*2010b* October/2010; 2015: August/2015; *DU13* domiciliar unit 13; *DU14* domiciliar unit 14, *W* wild; *s.d.* standard deviations; *AN* total number of allele

**Fig. 1:**
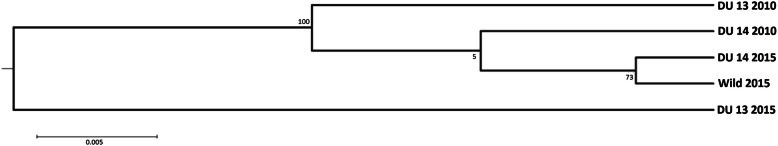
UPGMA dendrogram of the pairwise *FST* of *Triatoma brasiliensis*. Captured in domiciliary units (C13 and C14) and wild environment (S) of Cachoeira do Júlio (CJ) locality. Captured in 2010 and 2015. The numbers in the branch are the bootstrap

There were few events of Hardy-Weinberg disequilibrium when observed and expected heterozygosity values were considered (Table 7). The total variability was represented mainly by the diversity among all specimens (83.41%), followed by within-group variation (14.18%) and between-group variation (2.41%). The fixation indixes were: *F*_ST_ = 0.02 (*p* = 0.82); *F*_SC_ = 0.15 (*p* = 0.00); and *F*_CT_ = 0.17 (*p* = 0.00) (Table 1). The comparisons of pairwise *F*_ST_ showed that the group of DU13 (captured in 2010) differed from that in DU14 (captured in 2015), and also from the sylvatic sample. Bug captures in the two DUs in 2015 suggested no gene flow between them. The only group that presented inbreeding was from the sylvatic environment (*F*is with *p* < 0.05) (Table 8). Figure 1 shows the genetic relationship between groups. The reinfestation present in DU13 appears in an independent branch from the others. The most similar groups were the ones from the sylvatic environment and one from DU14, both captured in 2015.

**Table 7:** Observed (Ho) and expected (He) heterozygosity per locus of *Triatoma brasiliensis* captured in Cachoeira do Júlio locality

***locus***	Tb728		Tb830		Tb860		Tb8124		Tb7180	
Sample	Ho	He	Ho	He	Ho	He	Ho	He	Ho	He
**DU13_2010**	0.75	0.78	0.75	0.72	0.38	0.62	0.63*	**0.91**	0.75	0.72
**DU14_2010**	0.63	0.83	0.88	0.81	0.75*	**0.76**	0.75	0.88	0.63	0.77
**DU13_2015**	0.25*	**0.82**	0.60	0.73	1.00	0.80	0.40*	**0.64**	1.00	0.84
**DU14_2015**	0.38	0.51	0.75	0.78	0.88	0.74	0.63	0.87	0.75	0.90
**W_2015**	0.67	0.69	0.60	0.74	0.53	0.57	0.20*	**0.72**	0.80	0.74

*DU13* domiciliar unit 13; *DU14* domiciliar unit 14, *W* wild; 2010: October/2010; 2015: August/2015; *significant value (*p* < 0.05)

**Table 8:**
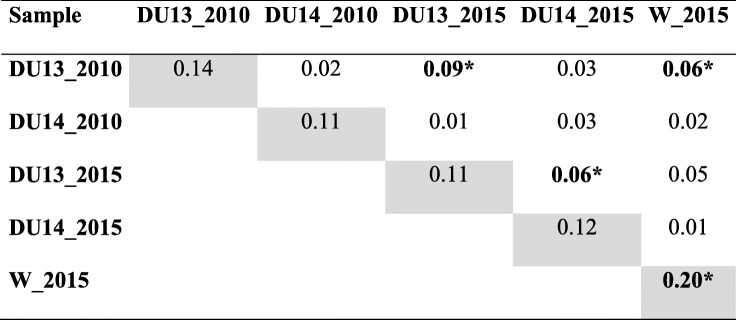
Genotypic differentiation among samples (pairwise *F*_ST_) and intra sample (*F*_IS_) in diagonal from Cachoeira do Júlio locality

*DU13* domiciliar unit 13; *DU14* domiciliar unit 14; *W* wild; 2010: October/2010; 2015: August/2015; *significant value (*p* < 0.05)

## Conclusions

The studied *T. brasiliensis* samples constitute a panmitic unit. The persistent infestation of DUs in Tauá may be happening for to two processes: active invasion of triatomines from their natural habitats and restoration of insect populations that survived after residual insecticide spraying, mainly related to operational failures in the process. Such processes explain the current challenges for vector surveillance and control, whose irregularity and limited coverage is a reality.

## Discussion

Microsatellite markers have been helpful for investigating the population dynamics of triatomines with the goal of designing more effective vector control strategies [23, 32, 35, 37–46]. Studies about gene flow can shed light on the process of domiciliation of autochthonous vectors such as *T. brasiliensis*. This species is adapted to human-made artificial ecotopes and the natural environment. Because of this characteristic, it is an epidemiologically important vector in the arid Northeastern region of Brazil [13, 43, 47, 48]. In the *caatinga*, the livelihoods of inhabitants and livestock production depend on local natural resources. Local inhabitants extract the products they need to survive in the semiarid land. Such process of occupation degrades and transforms the environment, as well as bridges the distance between human beings and various organisms [49], including *T. cruzi* hosts and vectors [13, 50].

The microsatellite *loci* used in this study were previously used for investigating the molecular ecology of *T. brasiliensis* in Rio Grande do Norte, Northeastern Brazil [23]. These authors described and used other three microsatellite markers and reported gene flow between domestic and sylvatic populations. Our current results for allele size are in agreement with the findings of previous studies [23, 28]. The *locus* Tb8112 was also monomorphic for *T. brasiliensis* in Rio Grande do Norte. Although a smaller number of *loci* was used, the samples from Ceará showed a higher average number of alleles when compared with those from Piauí, Bahia and Rio Grande do Norte [23], probably due to the larger number of individuals sampled.

In the three analyses performed, AMOVA indicated that total variation was mainly represented by individual differences. Variation between groups had a quite low representation, indicating that they were genetically similar, i.e., without population structure. *P* value of *F*_ST_ indicate little genetic variation among samples and corroborate the results mentioned above. This absence of clustering indicates that it is a panmictic unit, with free gene flow. Thus, it was important to verify whether the triatomines found after insecticide spraying also presented the same genetic structure. The large number of alleles with heterozygosis deficit is probably not due to the existence of null alleles, since the latter are not very likely to occur. The Hardy-Weinberg disequilibrium may have been caused by persistent infestation with specimens surviving insecticide spraying as well as the consequent increase of inbreeding. Its occurrence was evidenced by the statistically significant values of *F*is relative to groups 2009a, 2010b and 2015.

Although the global *F*_ST_values indicated lack of population structure, the pairwise comparison of this index provided further insights into population dynamics. There was no genotypic differentiation when the first four surveys were compared pairwise (*F*_ST_), except in the comparison between the first (February 2009) and the last one (October 2010). Such homogeneity can be explained by high gene flow of *T. brasiliensis* in this region, well-known for its great capacity of dispersal and colonization of domiciliary ecotopes [21, 37–40]. The difference between triatomines caught in the first and last surveys would reflect the elimination of preexisting triatomines following three insecticide applications (2009b, 2010a, 2010b) and the invasion of new specimens of *T. brasiliensis* into DUs over the 20-month period between these two surveys.

The dendrogram representing pairwise *F*_ST_of Cachoeira do Júlio samples makes it clear that microsatellites are highly sensitive markers for detailed analysis of population dynamics. Although geographically close (45 m) and interconnected by various peridomestic structures, the two DUs had different sources of infestation. DU 13 was probably reinfested after October 2010, given the clear separation of its two groups. The two catches at DU 14 were strongly associated with wild sample, especially the second one, although they were approximately 1300 m away. It may be concluded that this DU had a persistent infestation caused by residual foci and the continuous invasions of wild triatomines. This analysis also showed that *F*is was significant (*p* ≤ 0.05) in the wild sample. This fact suggests no flow of specimens from artificial to natural habitats.

Studies about gene flow can shed light on the process of domiciliation of autochthonous vectors such as *T. brasiliensis*. This species is adapted to human-made artificial ecotopes and the natural environment. Because of this characteristic, it is an epidemiologically important vector in the arid Northeastern region of Brazil [13, 38, 41, 42]. In the *Caatinga* biome, the livelihoods of inhabitants and livestock production depend on local natural resources. Local inhabitants extract the products they need to survive in the semiarid land. Such process of occupation degrades and transforms the environment, as well as bridges the distance between human beings and various organisms [43], including *T. cruzi* hosts and vectors [13, 44].

Microsatellite markers have been helpful for investigating the population dynamics of triatomines with the goal of designing more effective vector control strategies [23, 32, 35, 37, 41, 45–52]. Thus, the elimination of residual triatomine outbreaks after home spraying can be achieved if operational failures are identified and resolved, for example, through the correct application of insecticide, periodic coverage, supervision, monitoring and regular assessments of entomological and epidemiological indicators of worked areas. As for the prevention and early detection of invasions of wild insects, as it depends on the participation of residents, the intervention methodologies must be adequate considering the social, economic, ecological context and through an intersectoral approach that seeks sustainable, participatory and able to timely identify the risk of *T. cruzi* vector transmission infection in the human population [53, 54].

## Methods

### Research design and study site

**Fig. 2:**
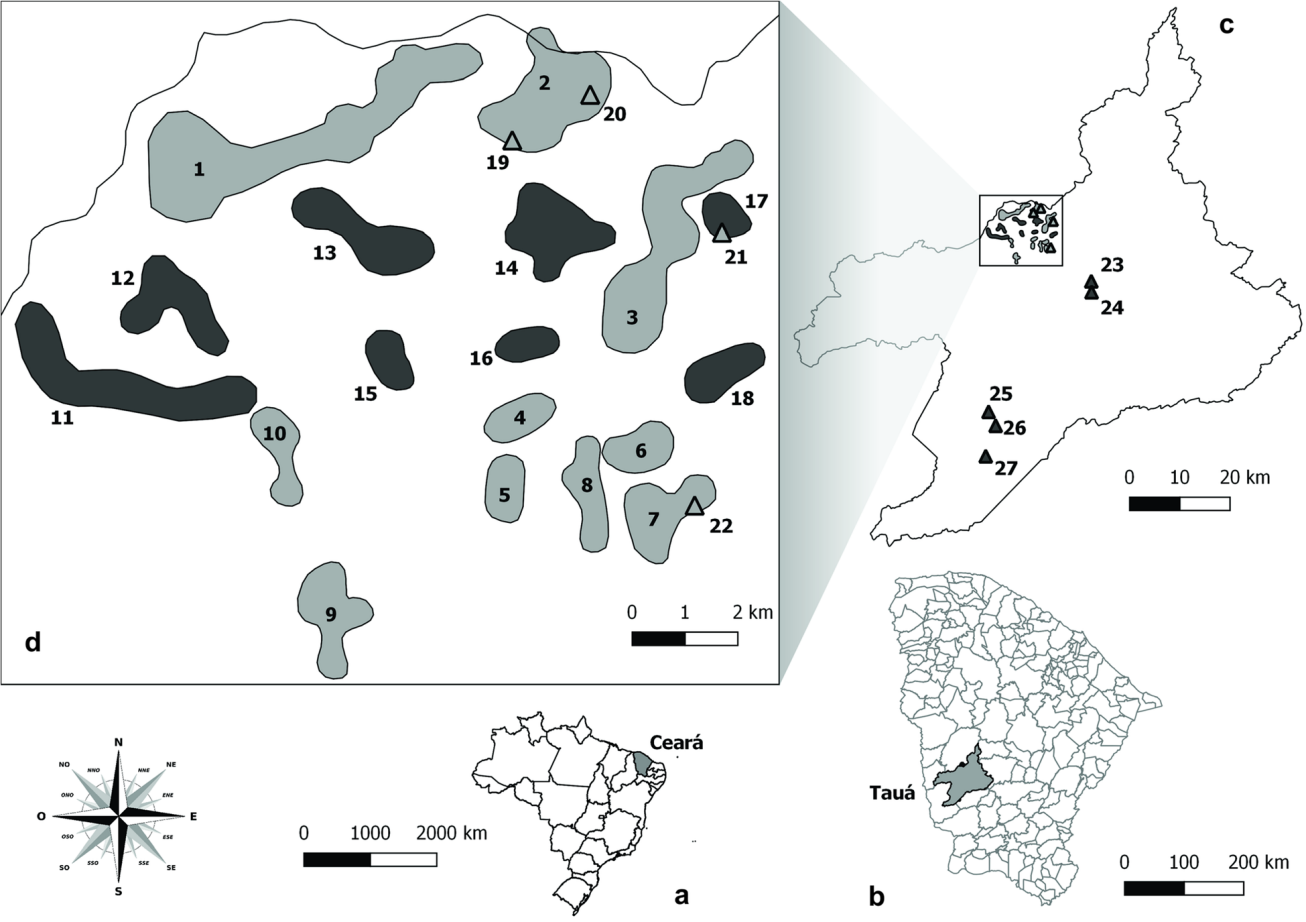
Location of study área. **a**. Location of Ceará State, Brazil. **b**. Area of Tauá munipality, Ceará, Brazil. **c**. Study site. **d**. Detail of study site. Gray polygons: general variability and variability along the time analysis: 1. Belo Horizonte do Alfredo; 2. Mutuca; 3. Cachoeira do Julio; 4. Benfica do INCRA; 5. Canadá; 6. Morada Nova do Pedro; 7. Morada Nova do Tomaz; 8. São Bento do INCRA; 9. São Cristóvão; 10. Várzea do Touro; triangles indicate wild environments: 19. Mutuca Evangelista; 20. Mutuca Pedra da Cruz; 21. Cachoeira do Julio; 22. Morada Nova do Tomaz. Black polygons: variability along the time analysis: 11. Santa Fé; 12. Merejo do Angico; 13. Açude Novo Sátiro; 14. Mutuquinha; 15. Jasmim do Aluísio; 16. Betânia; 17. Cachoeira dos Pedrosas; 18. Umburana; triangles indicate wild environments: 23. Juá; 24. Caraúbas; 25. Monte Cristo; 26. Aroeira; 27. Pedra D’água. Source: adapted from QGis 2.14. Essen

This is a longitudinal study conducted in the municipality of Tauá, in the arid Northeastern region of Brazil, in Ceará state (CE) (Fig. 2). Tauá is located in the Sertão dos Inhamuns (6°00′11″S; 40°17′34″W), 310 km from Fortaleza, at an altitude of 402.7 m. Average temperature ranges between 26 °C and 28 °C and average annual rainfall is 597.2 mm^3^, occurring from February to April [55]. Tauá is located in an area with deserts and xeric shrublands, where most trees and shrubs are thorny, dense, small and twisted, have small xeric leaves and well-developed roots. This vegetation is indicative of the most typical local climate. The presence of shallow, stony and dry soils during most of the year is a predominant characteristic [54, 56, 57]. Small mammals, reptiles and insects live in this habitat, including *T. brasiliensis*, whose natural biome is the *Caatinga* [58].

The Domiciliar Unit (DU) consists of human habitations (intradomicile) and its surroundings (peridomicile), including all permanent and temporary buildings, fences, piled materials, animal shelters, and so on [59]. The last residual insecticide spraying campaign against triatomines had occurred 2 years prior to the beginning of the present study. The DUs were inspected for infestation in five occasions: February 2009 (before first spraying), August 2009 (6 months after first spraying), April 2010 (14 months after first spraying), October 2010 (20 months after first spraying) and August 2015 (78 months after first spraying). Trained municipal health agents performed manual triatomines searches inside (intradomiciliar, minimum 30′) and around (peridomicile, minimum 30′) the house. All sighted triatomines were captured as much as possible, according to standard procedures [59]. A team from the State Department of Health supervised field activities was. All DUs (positive and negative) were sprayed with 20% SC alpha-cypermethrin (Fersol Industry and Trade) in Februay 2009, according to routine procedures of Chagas disease control program. In subsequent surveys only households with a current triatomine infestation were selectively sprayed with insecticide using the same procedures [60].

Sylvatic triatomines were searched at nine sites which had rocky outcrops, a typical natural ecotope of *T. brasiliensis*. Three of these sites were sampled in the five above-mentioned occasions; other six were included only in 2015 (Fig. 2, Table 9).

**Table 9:** Triatomines captured in the municipality of Tauá to evaluate the variability along the studied period

Period	February 2009				August 2009				April 2010				October 2010				August 2015				Total sample		
CL	DU	AE	WE	TT	DU	AE	WE	TT	DU	AE	WE	TT	DU	AE	WE	TT	DU	AE	WE	TT	AE	WE	insects
**ANS**																	1	15		15	15	0	15
**ARO**																			11	11	0	11	11
**Bet**																	1	1		1	1	0	1
**BHA**	1	2		2	1	1		1	1	2		2	5	11		11	1	4		4	20	11	31
**BI**					1	1		1	2	6		6	2	9		9	1	3		3	19	9	28
**Can**	1	1		1					2	4		4	1	5		5	2	2		2	12	5	17
**CB**																			12	12	0	12	12
**CJ**	2	3	17	20	5	13	16	29	1	1	29	30	5	22		22	7	31	15	46	70	99	169
**CP**									1	2		2					2	4		4	6	0	6
**JA**									1	2		2	1	1		1	2	4		4	7	1	8
**JUA**																			14	14	0	14	14
**M**	3	6		6					6	12		12	5	18		18	10	64		64	100	18	118
**MA**	1	2		2													3	20		20	22	0	22
**MC**																			12	12	0	12	12
**ME**			8	8			1	1			12	12							14	14	0	35	35
**Mnha**		1		1		1		1									3	11		11	13	0	13
**MNP**	5	14		14	2	2		2	3	3		3	2	4		4	4	7		7	30	4	34
**MNT**	2	2		2	2	2		2	4	5		5	2	3		3	10	15	11	26	27	14	41
**MPC**			32	32			15	15			13	13							7	7	0	67	67
**PA**																			15	15	0	15	15
**SBI**	1	5		5					2	2		2	2	2		2	2	2		2	11	2	13
**SC**					1	1		1	2	7		7					3	13		13	21	0	21
**SF**									1	1		1					2	5		5	6	0	6
**Um**									1	2		2					1	1		1	3	0	3
**VT**	1	5		5													1	5		5	10	0	10
**Total**	17	41	57	98	12	21	32	53	27	49	54	103	25	75		75	56	207	111	318	393	329	722

*CL* local of capture; *DU* Domiciliary units investigated; *EA* triatomines captured in artificial environments; *WE* triatomines captured in wild environments; *TT* total of triatomine; *ANS* Açude Novo do Sátiro; *ARO* Aroeira Bet Betânia; *BHA* Belo Horizonte do Alfredo; *BI* Benfinca do Incra; *Can* Canadá; CB:Caraúbas; *CJ* Cachoeira do Júlio; *CP* Cachoeira dos Pedrosas; *JA* Jasmim Aluizio; *JUA* Juá; *M* Mutuca; *MA* Merejo do Angico; *MC* Monte Cristo; *ME* Mutuca Evangelista; *Mnha* Mutuquinha; *MNP* Morada Nova do Pedro; *MNT* Morada Nova do Thomaz; *MPC* Mutuca Pedra da Cruz; *PA* Pedra D’Água; *SBI* São Bento do Incra; *SC* São Cristovão; *SF* Santa Fé; *Um* Umburana; *VT* Várzea do Touro

It was captured 3005 triatomines, being 64.2% in the artificial environment and 38.2% in the wild. For this study, we used samples with a minimum of five insects/group, as required for the molecular variance analysis (AMOVA) [61, 62].

Table 9 (stay here).

### Microsatellites analysis

#### General variability

For this analysis, 329 triatomines were used. The insects were captured on the four occasions in 2009 and 2010: 147 captured in 47 DUs e 182 in three rocky outcrops (Fig. 2; Table 3). The insects were obtained in 12 local of capture (10 only in artificial ecotopes, two in wild ecotopes and one in both environments) (Table 10). The insects were grouped according to the capture location regardless of their ecotope so that the largest number of locations could be represented due to the minimum sample size.

**Table 10:** *Triatoma brasiliensis* captured in the municipality of Tauá for the general variability study

Period	February 2009			August 2009			April 2010			October 2010			Total sample			
CL	DU	AE	WE	DU	AE	WE	DU	AE	WE	DU	AE	WE	DU	AE	WE	Total
**BHA**	1	2		1	2		1	2		5	11		6		17	17
**BI**				1	1		2	6		2	9		2		16	16
**Can**	1	1					2	4		1	5		2		10	10
**CJ**	2	3	18	5	13	16	1	1	29	5	23		6	63	40	103
**M**	3	6					6	12		5	19		11		37	37
**ME**			10			1			12					23	0	23
**MNP**	5	14		2	2		3	3		2	5		7		24	24
**MNT**	2	2		2	2		4	5		2	3		10		12	12
**MPC**			32			16			13					61	0	61
**SBI**	1	10								2	2		3		12	12
**SC**				2	2		2	7							9	9
**VT**	1	5													5	5
**Total**	16	43	60	13	22	33	21	40	54	24	77	0	47	147	182	329

*DU* Domiciliar units investigated; *EA* triatomines captured in artificial environments; *WE* triatomines captured in wild environments; *CL* local of capture; *BHA* Belo Horizonte do Alfredo; *BI* Benfica do Incra; *Can* Canadá; *CJ* Cachoeira do Júlio; *M* Mutuca; *ME* Mutuca Evangelista; *MNP* Morada Nova do Pedro; *MNT* Morada Nova do Thomaz; *MPC* Mutuca Pedra da Cruz; *SBI* São Bento do Incra; *SC* São Cristóvão; *VT* Várzea do Touro

#### Variability along the time

Triatomines from 25 locals of capture were clustered according to the five periods sampled, regardless of location and ecotope, in a total of 722 insects (Table 9).

#### Variability in Reinfestation in Cachoeira do Júlio

The peridomicile reinfestation of two DUs (13 and 14) in the locality of Cachoeira do Júlio was investigated in greater detail. For this analysis, the triatomines were grouped by DU and date of capture: (I) eight triatomines captured on October 2010 in DU 13; (II) five on August 2015 in DU 13; (III) eight on October 2010 in DU 14; (IV) eight on August 2015 on DU 14; and (V) 15 on August 2015 in the sylvatic environment.

#### Microsatellites genotyping

Genomic DNA was extracted from two legs of each specimen with the Wizard® Genomic DNA Purification Kit (Promega) following the manufacturer’s recommendations. DNA was quantified individually in a NanoDrop 1000 Spectrophotometer (Thermo Scientific) and stored at − 20 °C.

Primers were tested for nine microsatellite *loci* previously described for *T. brasiliensis*: Tb728, Tb830, Tb860, Tb7180, Tb8112, Tb8124 (13), Tb2146, Tb8102 and Tb8150 (11). Polymerase Chain Reactions (PCR) were run in a final volume of 10 μL containing 1 unit of Platinum® *Taq DNA polymerase (Invitrogen*), 1x buffer, 1.5 mM MgCl2, 1 mM dNTP, 5 pmol for each primer, 2 ng DNA and ultrapure water. The forward primers were labeled with a bioluminescent probe. The reactions were run in a Veriti® 96-Well thermocycler (*Thermo Fisher Scientific*) with the following cycle: initial denaturation at 95 °C for 5 min, 28 cycles at 94 °C for 30s, annealing at primer-dependent temperature for 30s, extension at 72 °C for 45 s, followed by a final extension at 72 °C for 5 min. Annealing temperatures were 48 °C for Tb860; 54 °C for Tb8112; 52 °C for Tb 2146; 56 °C for Tb8102; touchdown (reduction of incremental annealing temperature): 60 → 50 °C and 58 °C for Tb728, Tb830, Tb7180, Tb8124.

To determine locus size, the PCR products were diluted 1:10 in ultrapure water with a GeneScan™ 500 LIZ® dye size standard (*Thermo Fisher Scientific*) and genotyped in an ABI 3730 DNA Analyzer (*Applied Biosystem®*) by the DNA Sequencing Platform of René Rachou Institute. Chromatogram analysis was performed with the software Geneious 10.1.2© (*Biomatters Limited*).

#### Data Analysis

The number and size of alleles for each *locus*, observed (Ho) and expected heterozygosity (He) and Hardy-Weinberg equilibrium (HW) were determined using Arlequin version 3.1 [61, 62]. The occurrence of null alleles was checked with the software Micro-Checker 2.2.3; these frequencies were calculated for each locus by population with GENEPOP [63, 64]. Cluster presence was assessed by STRUCTURE [65].

AMOVA was used to determine the variance components and proportions of global, interpopulation and intrapopulation variability within total variation. In addition, the following fixation indices were estimated: *F*_ST_ (among populations), *F*_SC_ (among individuals within populations) and *F*_CT_ (within individuals). We evaluated the genotypic diversity between each sample pair (pairwise *F*_ST_) and intrapopulation (*F*_IS_). The tests were run with a 5% significance level and a maximum loss of 5% of amplified alleles. The Mantel test was also carry out in the cluster research. All these analyses were performed with Arlequin version 3.1 [61, 62]. To evaluated the reinfestation of Cachoeira do Júlio, a UPGMA tree also was built, based on genetic distance (pairwise *F*_ST_) (POPTREEW) [66].

## Supplementary information

**Additional file 1.**

**Additional file 2.**

**Additional file 3.**

## Data Availability

The data generated or analyzed in this study are included in this article and its additional files.

## Abbreviations

DU: Domiciliar unit
F_IS_: Inbreeding coefficient of an individual (I) relative to the subpopulation (S)
F_IT_: Inbreeding coefficient of an individual (I) relative to the total (T) population
FST: Inbreeding coefficient of subpopulations (S) compared to the total population (T)
HE: Heterozygosity Expected
HO: Heterozygosity Observed
HW: Hardy-Weinberg equilibrium
PCR: Polymerase Chain Reactions
STR: Short Tandem Repeats
UPGMA: Unweighted Pair Group Method With Arithmetic Mean
